# Controlling nutritional status score is a predictor of survival in hepatocellular carcinoma: A meta-analysis and meta-regression

**DOI:** 10.12669/pjms.41.4.11660

**Published:** 2025-04

**Authors:** Yi Chen, Hong Cao, Jia Yao

**Affiliations:** 1 Yi Chen, Department of Interventional Radiotherapy, Huzhou Central Hospital, Affiliated Central Hospital of HuZhou University, Huzhou, Zhejiang Province 313000, P.R. China; 2 Hong Cao, Department of Interventional Radiotherapy, Huzhou Central Hospital, Affiliated Central Hospital of HuZhou University, Huzhou, Zhejiang Province 313000, P.R. China; 3 Jia Yao, Department of Interventional Radiotherapy, Huzhou Central Hospital, Affiliated Central Hospital of HuZhou University, Huzhou, Zhejiang Province 313000, P.R. China

**Keywords:** Nutrition, Malnourished, Liver cancer, Survival

## Abstract

**Objective:**

The controlling nutritional status score (CONUT) has been utilized for prognostication of several cancers but its utility for hepatocellular carcinoma (HCC) is still unclear. We reviewed evidence on the ability of CONUT to predict overall survival (OS) and disease-free survival (DFS) in patients with HCC.

**Methods:**

Online repositories of PubMed, Embase, CENTRAL, and Web of Science were searched by two reviewers for English language studies and were available before 15^th^ March 2024. Hazard ratio (HR) and 95% confidence intervals were calculated for both OS and DFS.

**Results:**

A total of fourteen studies were available. Meta-analysis showed that CONUT was a significant predictor for OS (HR: 1.64 95% CI: 1.30, 2.06) and DFS (HR: 1.32 95% CI: 1.17, 1.50) in HCC. The effect size failed to change in significance on sensitivity analysis. Subgroup analysis based on country and treatment of HCC did not change the results. Subgroup analysis and meta-regression showed that a higher CONUT cut-off led to a stronger association between CONUT and OS in HCC.

**Conclusions:**

CONUT can be an easy-to-use and rapid prognostic indicator for HCC. High CONUT scores are associated with worse OS and DFS.

## INTRODUCTION

Liver cancer represents the 6^th^ most common malignancy and 3^rd^ common cause of cancer-related mortality in the world.^1^ Hepatocellular carcinoma (HCC) constitutes 75% of all liver cancer patients.^2^ Its incidence varies geographically with Asian and African populations being the largest contributors.^3^ Liver cirrhosis is the principal risk factor for HCC and traditionally viral diseases have been the biggest contributors.^4^ However, metabolic risk factors like non-alcoholic fatty liver disease, obesity, and metabolic syndrome are fast becoming the biggest contributors to HCC.^5^ Treatment of HCC is primarily surgical but early cases can be managed with radiofrequency (RFA) or microwave radiation. Also, transarterial chemoembolization (TACE) and transarterial radioembolization remain alternative therapies.^6^ Mortality with HCC remains high and there is a need for the identification of modifiable risk factors which can improve patient prognosis.

Traditionally, prognostic models for HCC have focused on cancer stage, tumor size, and distant metastasis.^7^ Malnutrition has now been recognized as a risk factor for poor survival, increased recurrence, and incomplete response to cancer therapy.^8^,^9^ However, assessment of malnutrition is a major problem.^10^ In this regard, several simple, easy-to-use, and rapid nutritional assessment tools like the prognostic nutritional index, controlling nutritional status score (CONUT), geriatric nutritional index have been developed.^11^ However, no single marker is gold-standard.

The CONUT score is a malnutrition assessment tool obtained from albumin, cholesterol, and absolute lymphocyte counts.^12^ It can predict the prognosis of various malignancies like colorectal,^13^ gastric,^14^ breast,^12^ and urinary bladder.^15^ However, its utility for HCC is still unclear. Previously, Takagi et al^16^ in a systematic review attempted to examine the efficacy of CONUT for predicting the prognosis of HCC but could include just four studies in the meta-analysis on survival. With newer studies in literature, there is a need for up-to-date evidence. Herein, we present the results of the most updated systematic review and meta-analysis examining the prognostic ability of CONUT for survival after HCC.

## METHODS

We conducted this systematic review and meta-analysis in concurrence with the PRISMA^17^ reporting guidelines. The review protocol was submitted to PROSPERO (CRD42024507179).

### Search protocol:

The online repositories of PubMed, Embase, CENTRAL, and Web of Science were searched for articles published in the English language and available before 15^th^ March 2024. Two search strings were used for all databases:

- (hepatocellular carcinoma) OR (liver cancer)) AND (controlling nutritional status) and
- (hepatocellular carcinoma) OR (liver cancer)) AND (CONUT). The bibliography of eligible articles and prior meta-analyses on the study topic were also scanned. We also searched ClinicalTrials.gov (www.clinicaltrials.gov) and Google Scholar to include ongoing studies and gray literature respectively.

All search results were combined in a single reference manager software (EndNote). Duplicates were eliminated. Two authors separately read the titles and abstracts of the articles to look for relevant studies. In the second phase, the two reviewers screened the full texts and excluded reports that did not meet the eligibility criteria. Disagreements were resolved in consultation with the third reviewer.

### Inclusion criteria:

- Cohort study design.
- Conducted on patients with HCC.
- Examined overall survival (OS) or disease-free survival (DFS) based on CONUT score. There was no restriction on the cut-off for CONUT.
- Reported outcomes as effect ratio with 95% confidence intervals (CI) or reported data to calculate the same.

### Exclusion Criteria:

- Studies published only as abstracts and non-peer-reviewed data.
- Studies not reporting required outcomes.
- Studies from the same institute with overlapping or duplicate data.

### Data management:

A pre-defined data collection form was used to collect the following data: study characteristics (author, year of publication, country, study design, sample size), patient characteristics (age, gender, Hepatitis-B virus [HBV] positive, Barcelona Clinic Liver Cancer stage, Child-Pugh grade, treatment received), CONUT details (cut-off, method to assess cut-off, number with high CONUT), follow-up, and outcomes. The primary outcome was OS, and the secondary outcome was DFS. Multivariable adjusted outcomes were extracted where available. If not reported, univariate data was to be used.

### Study quality:

Two reviewers assessed the quality of studies using the tailored form of the Newcastle-Ottawa Quality Assessment Scale (NOS).^18^ The final score of each study can range from 0 meaning the highest risk of bias up to Nine meaning the lowest risk of bias. The reviewers cleared all disagreements by discussion.

### Statistical analysis:

Data from the included studies was pooled to obtain hazard ratio (HR) and 95% CI in “Review Manager” (RevMan, version 5.3; Nordic Cochrane Centre (Cochrane Collaboration), Copenhagen, Denmark; 2014). A random-effects model was chosen. The chi-square test and the I^2^ statistic were also calculated to assess the heterogeneity between studies; I^2^>50% indicated substantial heterogeneity. Funnel plots were used to examine publication bias. Leave-one-out analysis was conducted in the software itself. Subgroup analysis was done for the following variables: country of the study, treatment of HCC, cut-off of CONUT, and method to obtain cut-off. Meta-regression was conducted using “metaHUN: a web tool for meta-analysis” (http://softmed.hacettepe.edu.tr/metaHUN/). The influence of sample size, age, male gender, HBV, Child-Pugh grade A, cut-off CONUT, and number with high CONUT was examined using the analysis.

## RESULTS

**Fig.1:**
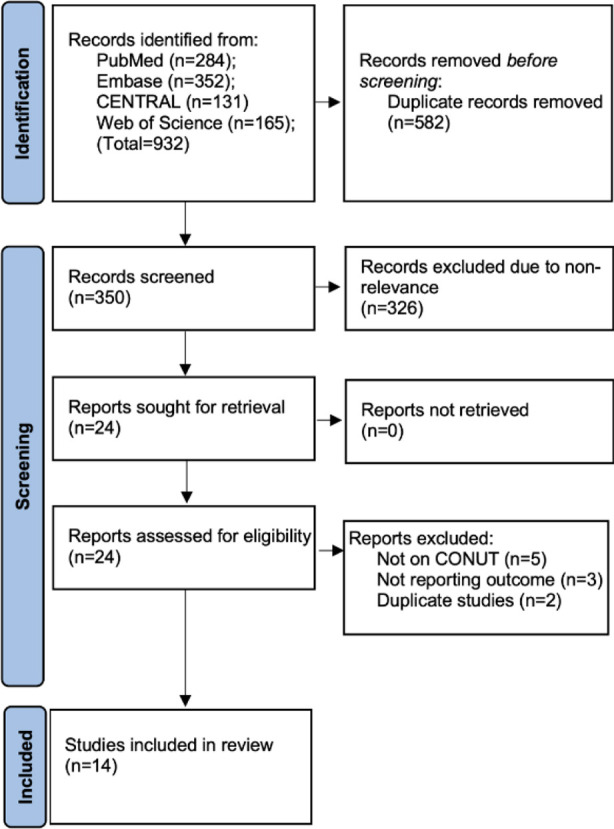
PRISMA flowchart demoting the workflow of the review.

We retrieved 932 studies from the four databases. On deduplication, 350 articles underwent initial screening and 24 underwent full-text analysis. Fourteen studies^19^,^20^–^29^,^32^ were finally selected for review. Fig.1.

All studies were retrospective and from China, Japan, or Germany (Table-I). A total of 6414 HCC patients were included in these studies. Patients were treated with surgery in most studies. Two studies included only patients receiving RFA, another two included those receiving TACE, while one study^25^ included patients with unresectable HCC treated with Lenvatinib. CONUT scores were calculated at the time of diagnosis or before initiating treatment in all studies. Most studies used predetermined cut-offs for CONUT while four studies used receiver operating characteristic curve. The cut-off used was either two, three, four or five. All studies reported multivariable adjusted data for OS and DFS except for Tamai et al^32^ who reported univariate data for DFS. The reviewers judged the studies based on NOS and awarded 7 to 9 points.

**Table-I:** Details of studies analyzed for meta-analysis.

Study	Type	Country	Sample size	Male gender	Age (years)	HBV + (%)	BCLC stage	Child-Pugh grade	Treatment	Cut-off for CONUT	High CONUT (%)	Cut-off method	Out-comes	FU*	NOS score
Tamai 2022^32^	R	Japan	181	129	71.4	NR	0: 75 A: 106	A: 157 B: 24	Surgery, RFA	≥3	40.3	Pre-determined	OS, DFS	39.2± 13.7 months	8
Qian 2022^31^	R	China	661	572	51	83.9	A: 360 B: 118 C: 172	A: 621 B: 40	Surgery	≥3	10.1	Pre-determined	OS, DFS	36 [19-39] months	7
Matsumoto 2022^30^	R	Japan	493	374	69	21.3	NR	A: 460 B: 37	Surgery	≥2	48.1	Pre-determined	OS	51.2 months	7
Chen 2022^29^	R	China	228	190	64.3	79.8	NR	A: 228	TACE	≥4	47.8	ROC	OS, DFS	41·7 (95% CI 39·2- 44·4) months	8
Tsunematsu 2021^19^	R	Japan	111	92	69	0	NR	A: 102 B: 9	Surgery	≥4	12.6	Pre-determined	OS, DFS	4.1 years [1.7–7.7] years	8
Peng 2021^28^	R	China	266	224	50	92.8	NR	NR	Surgery	≥3	62	Pre-determined	OS, DFS	34 months	9
Muller 2021^27^	R	Germany	237	40	70	10.1	A: 43 B: 126 C: 48 D: 20	A: 94 B: 94 C: 21	TACE	≥3	61.2	Pre-determined	OS	NR	7
Yang 2020^26^	R	China	403	329	NR	81.9	NR	A: 398 B: 5	RFA	≥5	19.6	X-tile software analysis	OS, DFS	NR	7
Shimose 2020^25^	R	Japan	164	134	73	19.5	A: 73 B: 71	A: 158 B: 6	Kinase inhibitors	≥5	21.3	Pre-determined	OS	NR	7
Lin 2020^24^	R	China	380	333	50	100	A: 236 B/C: 144	NR	Surgery	≥2	49.2	ROC	OS, DFS	NR	7
Chen 2020^23^	R	China	325	270	NR	84.6	NR	NR	RFA	≥5	19.1	ROC	OS, DFS	NR	7
Wang 2019^22^	R	China	209	172	NR	100	A: 126 B: 40 C: 43	A: 172 B: 37	Surgery	≥3	35.4	Pre-determined	OS, DFS	NR	7
Harimoto 2018^21^	R	Japan	2461	1785	69	NR	NR	A: 2300 B: 161	Surgery	≥4	21.9	ROC	OS, DFS	NR	7
Takagi 2017^20^	R	Japan	295	241	65.8	NR	NR	A: 288 B: 7	Surgery	≥3	40	Pre-determined	OS, DFS	42.3 months	8

R, retrospective; RFA, radiofrequency ablation; BCLC, Barcelona Clinic Liver Cancer; CONUT, controlling nutritional status; FU, follow-up; NR, not reported; NOS, Newcastle Ottawa scale; OS, overall survival; DFS, disease-free survival; TACE, transarterial chemoembolization; CI, confidence intervals; ROC, receiver operating characteristic; HBV+, Hepatitis-B virus positive

* As mean± standard deviation or median[interquartile range]

### Meta-analysis:

**Fig.2:**
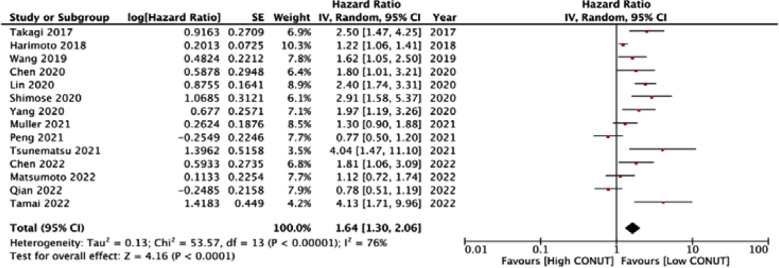
Forest plot for the association between CONUT and OS in HCC.

**Supp Fig.1:**
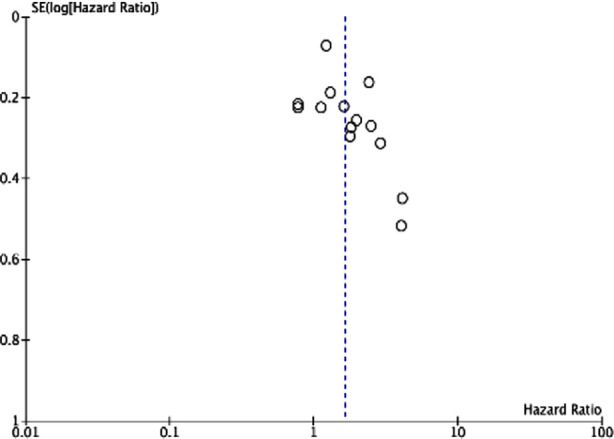
Funnel plot for the meta-analysis on OS.

Meta-analysis showed that CONUT was a significant predictor for OS in HCC (HR: 1.64 95% CI: 1.30, 2.06). Significant heterogeneity was noted (I^2^=76%) (Fig.2). No outliner was found on sensitivity analysis. No publication bias was noted. (Supp Fig.1).

**Fig.3:**
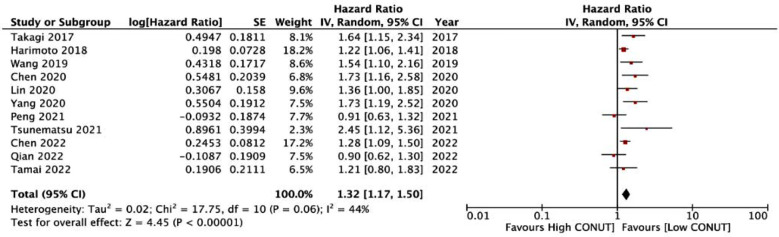
Forest plot for the association between CONUT and DFS in HCC.

**Supp Fig.2:**
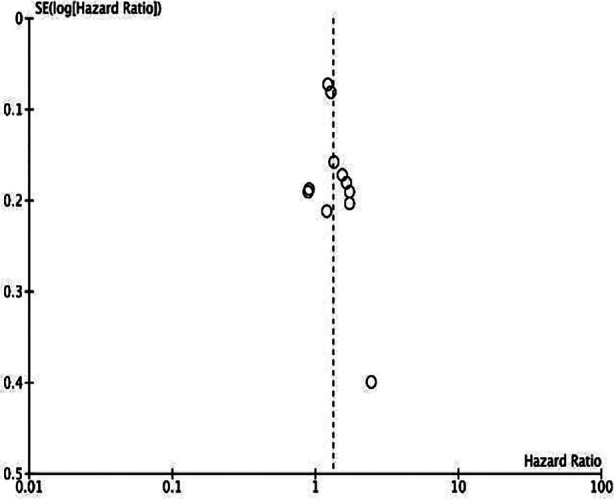
Funnel plot for the meta-analysis on DFS.

Meta-analysis also demonstrated that CONUT was a significant predictor for DFS in HCC (HR: 1.32 95% CI: 1.17, 1.50) Fig.3 Heterogeneity was not significant with I^2^=44%. No publication bias was noted (Supp Fig.2). There was no change in the results of the sensitivity analysis.

### Subgroup analysis and meta-regression:

Details of subgroup analysis for both OS and DFS are shown in Table-II. The results did not change in significance on subgroup analysis based on study location (China and Japan) and treatment modality. Subgroup analysis based on the method of determination of cut-off did not change the results of OS but the effect size turned non-significant for DFS (predetermined values). Further, on subgroup analysis based on the CONUT cut-off, it was noted that the results turned non-significant for lower cut-offs but remained significant for higher cut-offs.

**Table-II:** Subgroup analysis.

Variable	Groups	Studies	Hazard ratio [95% confidence intervals]	I^2^
OS				
Country	China Japan	7 6	1.47 [1.02, 2.12] 2.08 [1.33, 3.24]	79 80
	Germany	1	1.30 [0.90, 1.88]	-
Treatment	Surgery	8	1.44 [1.05, 1.96]	81
	RFA	2	1.89 [1.30, 2.77]	0
	TACE	2	1.45 [1.07, 1.96]	0
Cut-off	≥2	2	1.67 [0.79, 3.51]	87
	≥3	6	1.43 [0.93, 2.19]	79
	≥4	3	1.74 [1.01, 3.00]	71
	≥5	3	2.13 [1.55, 2.95]	0
Method of cut-off	Pre-determined	9	1.61 [1.12, 2.31]	78
	ROC	4	1.72 [1.15, 2.58]	81
DFS				
Country	China	7	1.30 [1.10, 1.55]	53
	Japan	4	1.37 [1.10, 1.71]	40
Treatment	Surgery	7	1.28 [1.06, 1.53]	53
	RFA	2	1.73 [1.32, 2.28]	0
	TACE	1	1.28 [1.09, 1.50]	-
Cut-off	≥2	1	1.36 [1.00, 1.85]	-
	≥3 ≥4	5 3	1.21 [0.94, 1.56] 1.28 [1.10, 1.48]	58 34
	≥5	2	1.73 [1.32, 2.28]	0
Method of cut-off	Pre-determined	6	1.28 [0.98, 1.66]	60
	ROC	4	1.28 [1.16, 1.41]	0

RFA, radiofrequency ablation; TACE, transarterial chemoembolization; OS, overall survival; DFS, disease-free survival.

Meta-regression analysis results are shown in Supp Table-I. For OS, the effect size for all moderators except for the cut-off was non-significant. Meta-regression showed a significant association between CONUT cut-off and effect size indicating that a higher cut-off led to a stronger association between CONUT and OS in HCC. For DFS, except for HBV positivity, no significant associations were noted. The analysis showed that the higher the HBV positivity rate, the stronger the association between CONUT and DFS in HCC.

## DISCUSSION

Our updated systematic review and meta-analysis examining the association between CONUT and outcomes of HCC showed that CONUT is an independent predictor of OS and DFS in HCC. Further, there seems to be a positive correlation between CONUT cut-off and OS with higher cut-offs associated with worse OS. No such relationship was noted for DFS. Since its first description in 2005,^33^ CONUT has become a widely used tool for malnutrition screening in cancer as well as non-cancer patients.^12^ Ulíbarri et al^33^ have shown that when compared to the standard Full Nutritional Assessment as a screening tool, CONUT has a sensitivity of 92% and specificity of 85% in detecting poor nutrition. Given that a Full Nutritional Assessment requires a detailed history, dietary intake, anthropometric examinations using sophisticated tools, and several laboratory values,^33^ the CONUT score with only three values significantly simplifies the assessment of malnutrition. However, to be routinely used in clinical practice, there must be strong evidence lending support to the prognostic utility of CONUT. Recently, CONUT has been recognized as a marker for survival in several gastrointestinal cancers. Liu et al^34^ in a pooled analysis of 19 studies have shown that CONUT is an independent predictor of OS, DFS, and complications in gastric cancer patients. Ma et al^35^ in a meta-analysis of seven studies found that CONUT predicted OS but not DFS in patients with pancreatic cancer. Jiang et al^36^ in a meta-analysis noted a positive association between CONUT and dismissal OS and worse DFS in biliary tract cancer.

However, data on HCC is limited to only one systematic review.^16^ Takagi et al^16^ in the previous review noted that CONUT was a predictor of OS and DFS in HCC but with only four studies in the analysis. Our meta-analysis presents a significant update to their review^16^ by including 10 more studies thereby improving the available evidence. In concurrence with previous results, we noted that CONUT was an independent predictor of OS and DFS with a 64% increased risk of mortality and a 32% increased risk of recurrence. The funnel plot showed that there were some studies indicating no value of CONUT in predicting OS or DFS. However, none of them were outliners, and the exclusion of any single study did not change the results.

Secondly, the inter-study heterogeneity for OS was quite high. In the subgroup analysis, much of the heterogeneity persisted indicating the role of unmeasured confounders which could have influenced outcomes. Nevertheless, we were able to examine the impact of important confounders like country of origin and treatment type on the effect size and noted persistent significant results. Meta-regression showed that the power of the study, patient demographics, Child-Pugh grade, and proportion of malnourished in the sample did not have a significant effect on the effect size. Important to note that on subgroup analysis and meta-regression, a stronger association of CONUT and survival was seen with higher cut-offs. This is plausible as a higher cut-off means increased severity of malnutrition which could lead to poor survival. Another positive association noted on meta-regression was between HBV positivity and worse DFS. The etiology of HCC has been one of the confounders influencing HCC survival but with mixed results.^37^ HBV has been shown to worsen DFS after hepatectomy indicating the need for adjuvant antiviral therapy.^38^ Since scarce data was available on DFS, there is a need for further research on the influence of viral etiology affecting the prognostic ability of CONUT.

The ability of CONUT to predict outcomes in HCC can be ascribed to its three elements. Albumin is a nutritional and systemic inflammatory response marker in patients with cancer.^39^ Since albumin is produced by the hepatocytes, it also reflects the baseline liver function. Reduced albumin represents cancer cachexia which is associated with poor survival, reduced response to treatment, and increased complications in HCC.^40^ȓ^42^ Patients with low albumin levels have high recurrence rates after HCC.^43^ Lymphocytes form a part of cell-mediated immunity and control the growth and metastasis of cancerous cells. By promoting apoptosis, they have a major role in immune surveillance of cancer. Tumor-infiltrating lymphocytes have a significant contribution to the development, prognosis, and response to immunotherapy treatment in HCC.^44^ Lastly, serum cholesterol indicates the patient’s nutrition and energy reserve. Individuals with low cholesterol are energy deficient with reduced cellular capacity to maintain metabolism, hormonal balance, and membrane integrity.^45^

### Limitations

Foremost, the predominance of Chinese and Japanese studies is a major hindrance to the generalizability of the results. More data is needed from Western populations. Secondly, all included studies focused only on pre-treatment measurements and there is no data on how changes in CONUT affect prognosis. Thirdly, several methodological differences were present among the included studies. Differences in patient characteristics, HCC stage, treatment, CONUT cut-off, and follow-up could potentially skew the results. Lastly, our review could only focus on OS and DFS. There is limited data in the literature on how CONUT affects treatment response to RFA or TACE or the risk of post-hepatectomy complications.

### Strengths of the review:

It include the updated literature search and inclusion of ten new studies as compared to previous review^16^ which significantly improves the available evidence. Oure review also has certain clinical implications. We have shown that CONUT can be used to predict survival after HCC and since it is a readily available marker, clinicians can easily incorporate in clinical practice for bed-side prognostication of HCC patients. However, there is a need for further studies to improve the quality of evidence specially to determine the optimal cut-off. Also, future studies should scrutinize the effect of nutritional support on intra-treatment and post-treatment CONUT scores and its influence on survival.

## CONCLUSIONS

CONUT can be an easy-to-use and rapid prognostic indicator for HCC. High CONUT scores are associated with worse OS and DFS. There seems to be a positive association between higher CONUT cut-off and dismissal OS after HCC.

### Authors’ contributions:

**YC:** Study design, literature search and manuscript writing.

**HC and JY:** Data collection, data analysis and interpretation. Critical Review.

**YC:** Was involved in the manuscript revision and validation.

All authors have read, approved the final manuscript and are responsible for the integrity of the study.
